# Growth, conformation, and egg-weight traits of CARI Nirbheek and CARI Shyama chickens across the foundation cross and first inter se generation

**DOI:** 10.3389/fvets.2026.1945142

**Published:** 2026-10-08

**Authors:** Jaydip Rokade, Roshni Chand, Nitish Gaitri, Vivek Srivastava, Gautham Kolluri, Darshana Bhaisare, Sai Siva Kumar Katam, Matin Ansari

**Affiliations:** 1 Indian Council of Agricultural Research (ICAR)-Central Avian Research Institute, Izatnagar, Bareilly, Uttar Pradesh, India; 2 Indian Council of Agricultural Research (ICAR)-Indian Veterinary Research Institute, Izatnagar, Bareilly, Uttar Pradesh, India; 3 Nagpur Veterinary College, Maharashtra Animal and Fishery Sciences University, Nagpur, Maharashtra, India

**Keywords:** backyard poultry, CARI Nirbheek, CARI Shyama, inter se breeding, native chicken

## Abstract

**Introduction:**

Improved native chicken lines are important for low-input poultry systems and rural livelihood security. This study evaluated the phenotypic performance of CARI Nirbheek (Aseel Peela × CARI Red) and CARI Shyama (Kadaknath × CARI Red) across the foundation cross (F_1_) and first inter se generation (F_2_).

**Methods:**

The F_1_ and F_2_ cohorts comprised 6,000 and 1,200 chicks, respectively. Body weight, bodyweight gain, morpho-conformation traits, and egg weight at 27 and 40 weeks were recorded and compared between the two lines within each cohort and descriptively across cohorts.

**Results:**

CARI Nirbheek showed a larger body-frame profile, with greater shank length, keel length, and breast angle at all measured ages. Body weight was age-dependent: CARI Shyama was heavier at selected early juvenile ages, whereas CARI Nirbheek was generally heavier from 8 weeks onward, reaching 1,285.19 ± 6.383 g in F_1_ and 1,236.66 ± 8.367 g in F_2_ at 20 weeks. Between-line egg-weight differences were significant in F_1_ but not in F_2_. At 20 weeks, body weight was virtually unchanged between cohorts in CARI Shyama but was modestly lower in F_2_. CARI Nirbheek, whereas morphoconformation and egg-weight traits showed comparatively similar mean values across cohorts.

**Discussion:**

Both lines exhibited distinct phenotypic profiles that may inform future selection strategies, subject to confirmation of heritability and genetic correlations in subsequent generations. Because F_1_ and F_2_ were produced in different hatching and rearing cycles, generation was confounded with year, season, batch, and selection history; therefore, these comparisons represent cohort-level phenotypic differences rather than direct evidence of genetic change, heterosis retention, genetic fixation, or long-term stability.

## 1 Introduction

Backyard poultry has an important role in household nutrition, supplementary income, livelihood security, and the sustainable utilization of indigenous chicken germplasm in India (1). The country produced approximately 149.11 billion eggs and 5.14 million tons of meat during 2024–25, with poultry contributing nearly 49% of total meat production (2). The 20th Livestock Census recorded 851.81 million poultry, including 317.07 million birds maintained under backyard systems, representing a substantial increase over the preceding census (2–4). Nevertheless, the present per-capita availability of eggs remains below the recommendation of the Indian Council of Medical Research (2). Indigenous and improved native chickens are therefore particularly relevant to rural poultry development because of their adaptability to local environments, suitability for scavenging and semi-intensive systems, consumer preference, and contribution to household livelihoods (1, 5–7).

Indian indigenous chickens exhibit considerable diversity in growth, body conformation, production, and adaptive characteristics. Aseel is characterized by a compact and muscular body, strong shanks, well-developed skeletal conformation, and relatively high body weight, although its egg-production potential is comparatively modest (8–10). Kadaknath, in contrast, is distinguished by fibromelanotic pigmentation, a comparatively smaller body frame, and a distinct growth and production profile (11). Differences between Aseel and Kadaknath have also been reported for body weight, shank measurements, age at sexual maturity, egg weight, and egg production (8, 9, 12, 13). These contrasting attributes make the two breeds useful parental resources for improving rural-poultry performance while retaining recognizable native-type characteristics.

Crossbreeding has long been used in rural-poultry improvement to combine the adaptability and locally preferred characteristics of indigenous chickens with improved growth or reproductive performance from selected stocks (14). Indian crossbred-evaluation studies demonstrate that performance depends strongly on the parental combination. Niranjan et al. (15) reported significant differences among improved backyard varieties in growth, sexual maturity, egg weight, and egg production, while Kundu et al. (16) observed cross-specific variation in growth and laying performance among Nicobari × Vanaraja F_1_ combinations under hot-humid conditions. Two- and three-way crosses developed for backyard production have similarly shown variation in growth, body conformation, and reproductive performance (17, 18). More recently, Rajaravindra et al. (19) reported substantial genotype-dependent variation in body weight, egg traits, and heterotic response among rural-poultry crossbreds, further emphasizing the need for cross-specific evaluation. Comparable variation has been reported among Aseel-, Kadaknath-, and Dahlem Red-derived crosses (20), while diallel and reciprocal-cross studies indicate that heterosis, general and specific combining ability, maternal effects, and reciprocal effects can contribute differently to growth and production traits (21–23). Similar variation in genetic and reciprocal effects has also been described for egg-production traits in crossbred chickens (24). Collectively, these findings support multi-trait evaluation of developing crossbred populations rather than assuming a uniform benefit of crossbreeding.

Within the public-sector programme for rural poultry improvement, the Indian Council of Agricultural Research-Central Avian Research Institute (ICAR-CARI), Izatnagar, developed CARI Nirbheek and CARI Shyama using indigenous male lines and CARI Red as the improved female line. CARI Nirbheek was derived from Aseel Peela males × CARI Red females, whereas CARI Shyama was derived from Kadaknath males × CARI Red females (25, 26). CARI Red has Dahlem Red inheritance and contributed improved productive potential, while Aseel Peela and Kadaknath contributed their respective native-type phenotypic attributes (25, 26). CARI Nirbheek has subsequently shown favorable growth and economic performance under backyard conditions (27). Direct and reciprocal combinations involving these genetic backgrounds have also shown differences in growth, body conformation, egg weight, and egg-quality traits (25, 28), indicating that parental background and cross direction can influence phenotypic performance.

The present study forms part of a broader National Livestock Mission-supported breeding programme in which the Aseel Peela × CARI Red and Kadaknath × CARI Red populations are being advanced through repeated inter se mating and phenotypic selection. In this programme, inter se mating refers to mating among selected individuals within each crossbred population to produce the subsequent generation. Following a F_1_ cross, segregation and recombination may alter the distribution and expression of phenotypic traits in F_2_. Chicken intercross studies have demonstrated generation-dependent variation in growth and linear body measurements after inter se mating (29) and across successive recombinant generations (30). Accordingly, similarity or divergence between F_1_ and F_2_ alone cannot establish directional genetic improvement, retained heterosis, genetic fixation, or true-breeding stability; such conclusions require evaluation across successive generations together with pedigree- or genomic-based estimation of additive, non-additive, maternal, and environmental components (31–34). Because the F_1_ and F_2_ populations in the present study were produced in different hatching and rearing cycles, generation could not be separated completely from year, season, batch, and selection history. The comparisons were therefore interpreted as descriptive cohort-level phenotypic contrasts rather than direct estimates of genetic change.

Against this background, the present study evaluated body weight, interval body-weight gain, morpho-conformation traits, and egg weight in CARI Nirbheek and CARI Shyama across the foundation cross (F_1_) and first inter se generation (F_2_). We hypothesized that the two lines would differ in age-specific growth, body conformation, and egg weight within each cohort and that the magnitude of within-line F_1_-F_2_ differences would vary among traits. The objectives were therefore to characterize line-associated phenotypic differences between CARI Nirbheek and CARI Shyama within each cohort and to describe phenotypic similarity or divergence between F_1_ and F_2_, thereby providing baseline information for subsequent genetic evaluation and selection in the ongoing breeding programme.

## 2 Materials and methods

### 2.1 Study site and ethical approval

The study was conducted at the experimental poultry breeding farm and associated analytical facilities of Indian Council of Agricultural Research—Central Avian Research Institute, Izatnagar, Bareilly, Uttar Pradesh, India (28°22′ N, 79°25′ E). All birds were maintained under standardized institutional poultry-management practices, including deep-litter housing, controlled brooding, *ad libitum* access to feed and water, age-specific diets, routine vaccination, and uniform husbandry procedures across generations, as detailed in Section 2.3. The study protocol was reviewed and approved by the Institutional Animal Ethics Committee (IAEC) of ICAR-Central Avian Research Institute, Izatnagar, India.

### 2.2 Parental stocks, mating design, and generational structure

CARI Nirbheek and CARI Shyama were developed at ICAR-CARI using Aseel Peela and Kadaknath males, respectively, crossed with CARI Red females, a line with Dahlem Red inheritance. The present study evaluated the foundation cross (F_1_) and first inter se generation (F_2_) of these two lines under the ongoing National Livestock Mission breeding programme.

The F_1_ population was generated using 100 breed-representative indigenous sires for each cross. Artificial insemination was used to produce the required progeny while maintaining broad representation of the parental population. A total of 6,000 day-old F_1_ chicks were produced, comprising approximately 3,000 CARI Nirbheek and 3,000 CARI Shyama chicks.

For production of the first inter se generation (F_2_), candidate F_1_ birds were screened under the ICAR-CARI breeding programme. Body weight was used as a primary selection criterion at 8 and 20 weeks of age, with approximate target body weights of 400 g at 8 weeks and 1.5 kg at 20 weeks. From the eligible population, 100 males and 600 females per line were retained for inter se mating while maintaining representation of the available families. In females, part-period egg-production records from 24 to 40 weeks of age were additionally considered during selection. Inter se mating was subsequently carried out within each respective crossbred population to produce the next generation. The resulting F_2_ cohort comprised 1,200 chicks, with 600 chicks per line.

Individual birds were identified using wing-band numbers during phenotypic recording. However, the analytical dataset available for the present study did not retain links between individual phenotypic records and their corresponding sire-dam family identifiers. Consequently, family effects could not be incorporated into the statistical model, and comparisons were performed at the overall line level within each cohort.

### 2.3 Housing, brooding, and dietary management

Birds from both cohorts were reared under a uniform institutional management protocol to minimize management-related variation. Chicks were brooded and reared in deep-litter floor pens bedded with dry wood shavings maintained at a depth of 5–7 cm. General flock birds were housed at a stocking density of approximately 100–110 birds per pen (providing 1.8–2.0 sq. ft of floor space per bird during rearing) across both cohorts. Consequently, birds were distributed across 28–30 replicate floor pens per line in the F_1_ cohort (3,000 birds per line) and six replicate floor pens per line in the F_2_ cohort (600 birds per line). Feed and clean drinking water were provided *ad libitum* throughout the experimental period, ensuring unrestricted access by replenishing feeders whenever consumed. Linear feeder space (5 cm/bird) and automatic nipple drinkers (one drinker per 10 birds) were allocated uniformly across all pens. Brooding temperature was maintained at approximately 35 °C during the first week after hatch and was gradually reduced by 2–3 °C per week until brooding was discontinued at 4 weeks of age. Birds were fed age-specific diets according to the institute's nutritional standards: starter ration from 0 to 4 weeks, containing 22% crude protein and 2,900 kcal ME/kg; grower ration from 5 to 16 weeks, containing 18% crude protein and 2,800 kcal ME/kg; and developer/layer ration from 17 weeks onward, containing 16% crude protein, 2,750 kcal ME/kg, and 3.5% calcium. A uniform vaccination schedule was followed in both cohorts, including vaccination against Marek's disease at hatch, Newcastle disease at 1 week with booster vaccination at 8 weeks, and infectious bursal disease at 2 and 4 weeks.

### 2.4 Phenotypic and performance measurements

Phenotypic and performance traits were recorded using the same measurement protocol in both cohorts. Traits included body weight, interval body-weight gain, morpho-conformation traits, and egg weight. Sample sizes differed among traits according to the recording design and are specified separately for each trait.

#### 2.4.1 Body weight

Individual live body weights were recorded with a calibrated electronic balance (±0.01 g) at day-old (0 week) and at 2, 4, 6, 8, 10, 12, 16, and 20 weeks of age. In the F_1_ cohort, a representative sample of 720 birds per line was weighed at each age. In the F_2_ cohort, the entire available cohort of 600 birds per line was weighed at each age. Thus, the difference in body-weight sample size reflected the recording design and available cohort size.

#### 2.4.2 Body weight gain

Body-weight gain for each growth interval was calculated for each individual bird as the difference between body weight recorded at the end of the interval and body weight recorded at the end of the preceding recording age. Thus, gains for 0–2, 2–4, 4–6, 6–8, 8–10, 10–12, 12–16, and 16–20 weeks were calculated from corresponding consecutive body-weight records. The body-weight gain analysis included 720 birds per line in F_1_ and 600 birds per line in F_2_.

#### 2.4.3 Morpho-conformation traits

Morpho-conformation traits comprising shank length, keel length, and breast angle were recorded at 6, 10, 14, and 20 weeks of age in 400 birds per line in each cohort. Shank length and keel length were measured using digital Vernier calipers, whereas breast angle was measured using a mechanical goniometer, following established procedures for linear body measurements in chickens (35, 36).

Shank length is defined as the length of the tarsometatarsus from the hock joint to the metatarsal pad (36). Keel length is the linear distance along the keel bone from its anterior chest end to its posterior extremity toward the abdominal region (36). Breast angle is the angle across the breast at the anterior region of the keel bone; for its measurement, the goniometer was positioned approximately 1 cm posterior to the anterior edge of the keel and its arms were adjusted against the left and right breast surfaces (36). Shank and keel lengths were expressed in millimeters, whereas breast angle was expressed in degrees. The same anatomical landmarks and measurement procedures were applied to both populations and cohorts.

#### 2.4.4 Egg weight

Egg weight was recorded from 100 hens per line in each cohort at 27 and 40 weeks of age. Eggs collected at each recording age were weighed individually using a calibrated electronic balance with a precision of ±0.01 g. These ages represent predefined egg-weight recording time points in the present study and were not designated as the onset of lay or peak egg production.

### 2.5 Statistical analysis

Data were summarized as mean and standard error (SE) for each line and cohort. Between-line differences between CARI Nirbheek and CARI Shyama were evaluated separately within each cohort and at each corresponding age or interval using Welch's two-sample *t*-test, which does not require equality of variances. For body weight, body-weight gain, morpho-conformation traits, and egg weight, the individual bird represented the observational unit.

To account for multiple between-line comparisons across corresponding ages or intervals, raw *P*-values were adjusted using the Benjamini–Hochberg false discovery rate (FDR) procedure. Both raw and FDR-adjusted *P*-values were reported, with adjusted *P* < 0.05 considered statistically significant after correction for multiple testing. The magnitude of between-line differences was quantified using Cohen's *d*, calculated from pooled standard deviations.

Within-line comparisons between the F_1_ and F_2_ cohorts were also performed at corresponding ages, intervals, or recording stages using Welch's two-sample *t*-test. The resulting *P*-values were adjusted using the Benjamini–Hochberg FDR procedure. Cohort-level differences were additionally described using percentage change relative to the F_1_ mean and Cohen's *d*. Because the F_1_ and F_2_ birds were produced in different hatching and rearing cycles, generation was confounded with year, season, batch, and selection history. Consequently, F_1_-F_2_ comparisons were interpreted as descriptive cohort-level phenotypic comparisons rather than as direct estimates of genetic change, heterosis retention, or genetic fixation.

Before analysis, datasets were screened for missing records, transcription errors, and biologically implausible observations. Suspected transcription errors were checked against the original recording sheets before correction. Extreme observations were examined but were not excluded solely on the basis of statistical outlier criteria. Distributional characteristics of the individual-level traits were also examined during data screening. Because of the relatively large sample sizes used for these comparisons, formal normality tests were not used as a pass–fail criterion, as such tests can be highly sensitive to minor departures from normality at large sample sizes. Some trait distributions showed departures from normality; therefore, strict normality was not assumed. Welch's two-sample *t*-test was retained because of its robustness to unequal variances and departures from normality in large samples, and statistical inference was interpreted together with effect sizes and FDR-adjusted *P*-values. Independence was considered at the individual-bird level for the traits analyzed. All statistical analyses were conducted in R version 4.3.1, R Foundation for Statistical Computing, Vienna, Austria. Data handling and tabulation were performed using the dplyr (37) and openxlsx (38) packages.

## 3 Results

### 3.1 Body weight

Age-wise body weights for both lines across the F_1_ and F_2_ cohorts are presented in Table 1. In the F_1_ cohort, CARI Nirbheek had a higher day-old body weight than CARI Shyama (32.61 ± 0.143 vs. 30.76 ± 0.118 g), corresponding to a 6.0% higher mean body weight (*P* < 0.001). At 2 weeks, the two lines showed virtually identical body weights (70.09 ± 0.449 vs. 70.12 ± 0.337 g; *P* = 0.957). CARI Shyama was heavier at 4 and 6 weeks by 3.8 and 3.2%, respectively (*P* = 0.022 and *P* = 0.008), whereas CARI Nirbheek showed higher mean body weights from 8 through 20 weeks; age-wise means are given in Table 1. At 20 weeks, CARI Nirbheek weighed 1,285.19 ± 6.383 g compared with 1,178.94 ± 9.079 g for CARI Shyama, representing a 9.0% higher mean body weight (*P* < 0.001).

**Table 1:** Body weight (g) of CARI Nirbheek and CARI Shyama across F_1_ and F_2_ cohorts.

Age	F1				F_2_			
	Nirbheek	Shyama	*P*-value (raw/FDR)	Effect size (Cohen's *d*)	Nirbheek	Shyama	*P*-value (raw/FDR)	Effect size (Cohen's *d*)
Day-old	32.61 ± 0.143^a^	30.76 ± 0.118^b^	< 0.001/ < 0.001	0.53	32.57 ± 0.155	32.62 ± 0.447	0.922/0.972	−0.01
2 weeks	70.09 ± 0.449	70.12 ± 0.337	0.957/0.957	−0.00	77.83 ± 0.351^a^	71.03 ± 0.328^b^	< 0.001/ < 0.001	0.82
4 weeks	144.35 ± 1.493^b^	149.78 ± 1.845^a^	0.022/0.029	−0.12	151.05 ± 0.423^b^	157.29 ± 0.311^a^	< 0.001/ < 0.001	−0.69
6 weeks	219.79 ± 1.838^b^	226.73 ± 1.861^a^	0.008/0.012	−0.14	264.56 ± 0.995	264.52 ± 0.468	0.972/0.972	0.00
8 weeks	403.37 ± 3.048^a^	350.26 ± 2.421^b^	< 0.001/ < 0.001	0.72	435.28 ± 1.820^a^	357.31 ± 0.958^b^	< 0.001/ < 0.001	2.19
10 weeks	446.60 ± 3.323^a^	402.18 ± 3.069^b^	< 0.001/ < 0.001	0.52	616.37 ± 1.556^a^	467.44 ± 0.547^b^	< 0.001/ < 0.001	5.21
12 weeks	631.52 ± 4.226^a^	598.66 ± 8.402^b^	< 0.001/ < 0.001	0.18	940.49 ± 1.325^a^	899.50 ± 2.669^b^	< 0.001/ < 0.001	0.79
16 weeks	921.73 ± 2.986	899.20 ± 11.198	0.052/0.059	0.10	1,085.79 ± 8.537^a^	904.30 ± 6.196^b^	< 0.001/ < 0.001	0.99
20 weeks	1,285.19 ± 6.383^a^	1,178.94 ± 9.079^b^	< 0.001/ < 0.001	0.50	1,236.66 ± 8.367^a^	1,187.57 ± 10.368^b^	< 0.001/ < 0.001	0.21

Values are mean ± SE. F_1_: n = 720 birds per line; F_2_: n = 600 birds per line. Raw and BH-FDR-adjusted *P* values are shown as raw/FDR. BH-FDR correction was applied within each cohort across the nine age-wise between-line comparisons. Cohen's d is positive when CARI Nirbheek > CARI Shyama and negative when CARI Nirbheek < CARI Shyama. Different superscripts within a cohort indicate a statistically significant between-line difference at FDR-adjusted *P* < 0.05.

For the F_2_ cohort, no significant difference was observed between the two lines at day-old (32.57 ± 0.155 g vs. 32.62 ± 0.447 g; *P* = 0.922) or at 6 weeks (*P* = 0.972). CARI Nirbheek was heavier at 2 weeks (77.83 ± 0.351 g vs. 71.03 ± 0.328 g; *P* < 0.001), while CARI Shyama recorded higher body weight at 4 weeks (157.29 ± 0.311 g vs. 151.05 ± 0.423 g; *P* < 0.001). From 8 weeks through 20 weeks, CARI Nirbheek was significantly heavier at all ages (*P* < 0.001; Table 1). Final body weights at 20 weeks were 1,236.66 ± 8.367 g and 1,187.57 ± 10.368 g for CARI Nirbheek and CARI Shyama, respectively (*P* < 0.001).

### 3.2 Body weight gain

Body-weight gain showed clear interval-specific differences between CARI Nirbheek and CARI Shyama across the F_1_ and F_2_ cohorts (Table 2). In F_1_, CARI Shyama showed greater gain during 0–2 weeks (39.35 ± 0.36 vs. 37.48 ± 0.47 g; raw *P* = 0.002, FDR-adjusted *P* = 0.004), whereas CARI Nirbheek showed greater gain during 6–8 weeks (183.58 ± 3.53 vs. 123.53 ± 2.93 g; adjusted *P* < 0.001) and 16–20 weeks (363.54 ± 6.74 vs. 279.73 ± 14.08 g; adjusted *P* < 0.001). The difference during 2–4 weeks was significant before adjustment but did not remain significant after FDR correction (raw *P* = 0.026; adjusted *P* = 0.051). No significant between-line differences were observed during 4–6, 8–10, 10–12, or 12–16 weeks after FDR correction.

**Table 2:** Interval-wise body-weight gain (g) of CARI Nirbheek and CARI Shyama across F_1_ and F_2_ cohorts.

Interval (weeks)	F_1_				F_2_			
	Nirbheek	Shyama	*P*-value (raw/FDR)	Effect size (Cohen's *d*)	Nirbheek	Shyama	*P*-value (raw/FDR)	Effect size (Cohen's *d*)
0–2	37.48 ± 0.47^b^	39.35 ± 0.36^a^	0.002/0.004	−0.17	45.26 ± 0.38^a^	38.41 ± 0.55^b^	< 0.001/ < 0.001	0.59
2–4	74.26 ± 1.54	79.67 ± 1.87	0.026/0.051	−0.12	73.22 ± 0.53^b^	86.26 ± 0.44^a^	< 0.001/ < 0.001	−1.09
4–6	75.44 ± 2.27	76.95 ± 2.64	0.665/0.665	−0.02	113.51 ± 1.10^a^	107.23 ± 0.56^b^	< 0.001/ < 0.001	0.29
6–8	183.58 ± 3.53^a^	123.53 ± 2.93^b^	< 0.001/ < 0.001	0.69	170.72 ± 2.04^a^	92.80 ± 1.09^b^	< 0.001/ < 0.001	1.94
8–10	43.23 ± 4.44	51.92 ± 3.85	0.139/0.223	−0.08	181.09 ± 2.42^a^	110.13 ± 1.12^b^	< 0.001/ < 0.001	1.53
10–12	184.91 ± 5.52	196.48 ± 8.94	0.271/0.362	−0.06	324.12 ± 2.05^b^	432.05 ± 2.66^a^	< 0.001/ < 0.001	−1.85
12–16	290.22 ± 5.04	300.54 ± 14.07	0.490/0.560	−0.04	145.30 ± 8.61^a^	4.80 ± 6.85^b^	< 0.001/ < 0.001	0.74
16–20	363.54 ± 6.74^a^	279.73 ± 14.08^b^	< 0.001/ < 0.001	0.28	150.87 ± 12.11^b^	283.27 ± 11.84^a^	< 0.001/ < 0.001	−0.45

Values are mean ± SE. F_1_: n = 720 birds per line; F_2_: n = 600 birds per line. Raw and BH-FDR-adjusted *P* values are shown as raw/FDR. Cohen's d is positive when CARI Nirbheek > CARI Shyama and negative when CARI Nirbheek < CARI Shyama. Different superscripts within a cohort indicate a statistically significant between-line difference at FDR-adjusted *P* < 0.05.

In F_2_, all eight interval-wise between-line comparisons remained significant after FDR correction. CARI Nirbheek showed greater gains during 0–2, 4–6, 6–8, 8–10, and 12–16 weeks, whereas CARI Shyama showed greater gains during 2–4, 10–12, and 16–20 weeks. The largest standardized differences were observed during 6–8 weeks, when CARI Nirbheek gained 170.72 ± 2.04 g compared with 92.80 ± 1.09 g for CARI Shyama (Cohen's *d* = 1.94), and during 10–12 weeks, when CARI Shyama gained 432.05 ± 2.66 g compared with 324.12 ± 2.05 g for CARI Nirbheek (*d* = −1.85). A large difference was also observed during 8–10 weeks, with higher gain in CARI Nirbheek (181.09 ± 2.42 vs. 110.13 ± 1.12 g; *d* = 1.53).

### 3.3 Morpho-conformation traits

CARI Nirbheek consistently exhibited a larger conformation profile than CARI Shyama across both cohorts. In the F_1_ cohort, at 20 weeks, shank length was 91.61 ± 0.133 mm in CARI Nirbheek vs. 83.00 ± 0.184 mm in CARI Shyama, representing a 10.4% greater value. Keel length was 99.48 ± 0.168 vs. 93.91 ± 0.141 mm (5.9% greater), while breast angle was 60.09 ± 0.122° vs. 57.81 ± 0.058° (3.9% greater; *P* < 0.001 for all comparisons). Age-wise morpho-conformation measurements for both lines across the F_1_ and F_2_ cohorts are presented in Table 3.

**Table 3:** Morpho-conformation traits of CARI Nirbheek and CARI Shyama across F_1_ and F_2_ cohorts.

Trait	Age (weeks)	F_1_				F_2_			
		Nirbheek	Shyama	*P*-value (raw/FDR)	Effect size (Cohen's *d*)	Nirbheek	Shyama	*P*-value (raw/FDR)	Effect size (Cohen's *d*)
Shank length (mm)	6	39.07 ± 0.236^a^	34.30 ± 0.140^b^	< 0.001/ < 0.001	1.23	39.74 ± 0.226^a^	34.39 ± 0.142^b^	< 0.001/ < 0.001	1.41
	10	58.17 ± 0.298^a^	41.96 ± 0.112^b^	< 0.001/ < 0.001	3.60	57.96 ± 0.285^a^	41.95 ± 0.112^b^	< 0.001/ < 0.001	3.70
	14	84.34 ± 0.135^a^	74.24 ± 0.164^b^	< 0.001/ < 0.001	3.37	84.28 ± 0.134^a^	74.30 ± 0.161^b^	< 0.001/ < 0.001	3.37
	20	91.61 ± 0.133^a^	83.00 ± 0.184^b^	< 0.001/ < 0.001	2.68	91.56 ± 0.132^a^	83.01 ± 0.171^b^	< 0.001/ < 0.001	2.80
Keel length (mm)	6	44.53 ± 0.155^a^	43.10 ± 0.130^b^	< 0.001/ < 0.001	0.50	44.49 ± 0.160^a^	43.01 ± 0.131^b^	< 0.001/ < 0.001	0.51
	10	66.84 ± 0.293^a^	64.25 ± 0.207^b^	< 0.001/ < 0.001	0.51	66.15 ± 0.289^a^	64.24 ± 0.212^b^	< 0.001/ < 0.001	0.38
	14	80.18 ± 0.168^a^	78.08 ± 0.124^b^	< 0.001/ < 0.001	0.71	80.13 ± 0.170^a^	77.97 ± 0.122^b^	< 0.001/ < 0.001	0.73
	20	99.48 ± 0.168^a^	93.91 ± 0.141^b^	< 0.001/ < 0.001	1.80	99.43 ± 0.170^a^	93.85 ± 0.135^b^	< 0.001/ < 0.001	1.82
Breast angle (°)	6	25.09 ± 0.163^a^	23.73 ± 0.187^b^	< 0.001/ < 0.001	0.39	24.64 ± 0.152^a^	23.42 ± 0.185^b^	< 0.001/ < 0.001	0.36
	10	39.01 ± 0.129^a^	37.17 ± 0.125^b^	< 0.001/ < 0.001	0.72	38.76 ± 0.138^a^	37.20 ± 0.120^b^	< 0.001/ < 0.001	0.60
	14	47.37 ± 0.065^a^	45.39 ± 0.097^b^	< 0.001/ < 0.001	1.21	47.40 ± 0.066^a^	45.64 ± 0.089^b^	< 0.001/ < 0.001	1.12
	20	60.09 ± 0.122^a^	57.81 ± 0.058^b^	< 0.001/ < 0.001	1.19	59.95 ± 0.129^a^	57.86 ± 0.057^b^	< 0.001/ < 0.001	1.05

Values are mean ± SE (n = 400 birds per line in each cohort). Raw and BH-FDR-adjusted *P* values are shown as raw/FDR. Cohen's d is positive when CARI Nirbheek > CARI Shyama and negative when CARI Nirbheek < CARI Shyama. Different superscripts within a cohort indicate a statistically significant between-line difference at FDR-adjusted *P* < 0.05.

A similar pattern was observed in the F_2_ cohort. At 20 weeks, shank length was 91.56 ± 0.13 vs. 83.01 ± 0.17 mm (10.3% greater), keel length was 99.43 ± 0.16 vs. 93.85 ± 0.13 mm (5.9% greater), and breast angle was 59.95 ± 0.12° vs. 57.86 ± 0.05° (3.6% greater) in CARI Nirbheek and CARI Shyama, respectively (*P* < 0.001; Table 3).

### 3.4 Egg weight

Egg weights at 27 and 40 weeks of age for both lines across the F_1_ and F_2_ cohorts are given in Table 4. In the F_1_ cohort, egg-weight differences were small and changed direction between recording ages. At 27 weeks, CARI Shyama had a higher mean egg weight than CARI Nirbheek (41.03 ± 0.316 vs. 39.89 ± 0.318 g), corresponding to an approximately 2.9% difference (raw *P* = 0.012; FDR-adjusted *P* = 0.024). At 40 weeks, CARI Nirbheek had a slightly higher mean egg weight than CARI Shyama (51.03 ± 0.150 vs. 50.54 ± 0.181 g; approximately 1.0%; raw *P* = 0.040; FDR-adjusted *P* = 0.040).

**Table 4:** Egg weight (g) of CARI Nirbheek and CARI Shyama at 27 and 40 weeks across F_1_ and F_2_ cohorts.

Age	F_1_				F_2_			
	Nirbheek	Shyama	*P*-value (raw/FDR)	Effect size (Cohen's *d*)	Nirbheek	Shyama	*P*-value (raw/FDR)	Effect size (Cohen's *d*)
27 weeks	39.89 ± 0.318^b^	41.03 ± 0.316^a^	0.012/0.024	−0.36	39.91 ± 0.322	40.69 ± 0.314	0.085/0.140	−0.24
40 weeks	51.03 ± 0.150^a^	50.54 ± 0.181^b^	0.040/0.040	0.29	50.93 ± 0.190	50.50 ± 0.219	0.140/0.140	0.21

Values are mean ± SE (n = 100 hens per line in each cohort). Raw and BH-FDR-adjusted *P* values are shown as raw/FDR. BH-FDR correction was applied within each cohort across the two recording ages. Cohen's d is positive when CARI Nirbheek > CARI Shyama and negative when CARI Nirbheek < CARI Shyama. Different superscripts within a cohort indicate a statistically significant between-line difference at FDR-adjusted *P* < 0.05.

In the F_2_ cohort, the between-line differences were smaller and not statistically significant. At 27 weeks, egg weight was 39.91 ± 0.322 g in CARI Nirbheek and 40.69 ± 0.314 g in CARI Shyama (approximately 2.0% higher in CARI Shyama; raw *P* = 0.085, FDR-adjusted *P* = 0.140), whereas at 40 weeks it was 50.93 ± 0.190 and 50.50 ± 0.219 g, respectively (approximately 0.9% higher in CARI Nirbheek; *P* = 0.140; Table 4).

Thus, line differences in egg weight were modest and age-dependent in the F_1_ cohort and were not statistically significant in the F_2_ cohort.

### 3.5 Phenotypic similarity across F_1_ and F_2_ cohorts

Within-line comparisons between the F_1_ and F_2_ cohorts are presented in Supplementary Tables S1–S5. At 20 weeks, CARI Shyama showed very similar body-weight means between F_1_ and F_2_ (1,178.94 vs. 1,187.57 g; +0.7%; adjusted *P* = 1.000), whereas CARI Nirbheek showed a modest reduction from 1,285.19 to 1,236.66 g (−3.8%; Cohen's *d* = 0.26; adjusted *P* < 0.001). In contrast, body weight differed more markedly between cohorts at several intermediate ages, particularly at 12 weeks, when F_2_ values were higher than F_1_ values by 48.9% in CARI Nirbheek and 50.3% in CARI Shyama (Supplementary Table S1).

Morpho-conformation traits showed broadly similar mean values across the F_1_ and F_2_ cohorts. All 24 within-line trait-age comparisons for shank length, keel length, and breast angle were non-significant after false discovery rate correction. In particular, the 10-week shank-length means in CARI Shyama were virtually identical between F_1_ and F_2_ (41.96 vs. 41.95 mm), consistent with the overall phenotypic similarity of the conformation traits across cohorts. Keel length and breast angle likewise showed only small cohort-level differences across the measured ages (Supplementary Tables S2–S4).

Egg-weight means at both 27 and 40 weeks were also similar between the F_1_ and F_2_ cohorts within each line, with only small percentage changes between cohorts and no significant within-line differences after FDR correction (Supplementary Table S5). Overall, morpho-conformation and egg-weight traits showed comparatively similar phenotypic mean values across cohorts, whereas body weight exhibited greater cohort-level variation at several intermediate ages and a modest reduction in 20-week body weight in CARI Nirbheek.

## 4 Discussion

The present study demonstrated marked age-dependent differences in growth between CARI Nirbheek and CARI Shyama across the foundation cross and first inter se generation. Rather than showing uniform superiority of either line throughout development, the relative ranking changed with age. In F_1_, CARI Shyama was heavier at 4 and 6 weeks, whereas the lines were similar at 2 weeks; in F_2_, CARI Nirbheek was heavier at 2 weeks, CARI Shyama at 4 weeks, and no difference was evident at 6 weeks. From 8 weeks onward, however, CARI Nirbheek generally exhibited the higher body weight in both cohorts. The interval-wise body-weight-gain data reinforce this interpretation, as the line showing the greater gain differed among growth intervals, particularly in F_2_. Thus, the principal distinction between the populations appears to involve the timing and distribution of growth rather than a uniformly greater growth rate in one line. Age-dependent variation in growth is well-recognized in chickens because growth rate, mature body size, and the expression of body weight depend on both genetic background and age of measurement (39, 40).

The later-age advantage of CARI Nirbheek is biologically consistent with its Aseel background. Aseel has generally been reported to attain greater body weight and possess a larger skeletal frame than Kadaknath (8, 9, 12), while comparatively slower early growth followed by greater later growth has also been described in Aseel (10). Haunshi et al. (41) similarly reported significantly greater juvenile body weights in Aseel than Kadaknath, although the early-age ranking observed in the present crossbred lines did not simply reproduce that of the parental breeds. This illustrates that crossbred growth trajectories cannot be inferred directly from parental means. Comparable genotype- and age-dependent differences have been observed among improved backyard varieties (15) and among crossbreds developed for rural poultry production (17). More directly, Aseel and Kadaknath derived crosses have shown age-dependent differences in body weight (20, 35), and Pal et al. (28) reported significant growth variation among direct and reciprocal Aseel Peela/Kadaknath × CARI Red combinations. Collectively, these findings support the interpretation that the contrasting growth trajectories of CARI Nirbheek and CARI Shyama reflect cross-specific phenotypic expression associated with their different parental backgrounds rather than uniform superiority of either population. Physiological mechanisms underlying these differences, such as endocrine regulation, metabolic rate, or muscle accretion, were not evaluated and therefore cannot be inferred from the present data.

The morpho-conformation results provide a more consistent distinction between the two populations. CARI Nirbheek exhibited greater shank length, keel length, and breast angle at every measured age in both cohorts, indicating a persistently larger body-frame phenotype. At 20 weeks, the between-line difference was particularly evident for shank length, which was approximately 10% greater in CARI Nirbheek in both cohorts, while keel length and breast angle were also consistently greater. This pattern agrees with descriptions of Aseel as a heavier, longer-legged and more strongly built indigenous breed than Kadaknath (8, 9, 12). Haunshi et al. (41) recorded greater 6-week shank length in Aseel than Kadaknath (57.43 vs. 51.46 mm) and reported strong positive genetic and phenotypic associations between body weight and shank length in both breeds. Morphometric analysis has likewise demonstrated close relationships between body weight and body measurements in Aseel and Kadaknath (42). Similar cross-specific variation in shank and keel dimensions has been reported among rural-poultry crossbreds (17), while Pal et al. (28) observed significant differences in shank length, keel length, and breast angle among direct and reciprocal Aseel Peela/Kadaknath × CARI Red crosses. The agreement between parental-breed and related crossbred evidence therefore supports a distinct larger-frame phenotype of CARI Nirbheek. Nevertheless, these measurements represent line-associated phenotypic differences; without pedigree-linked records and genetic variance-component analysis, they should not be interpreted as estimates of genetic response.

Interpretation of differences between F_1_ and F_2_ requires greater caution. At 20 weeks, the between-line body-weight separation decreased from approximately 9.0% in F_1_ to about 4.1% in F_2_, but this reduction cannot by itself be attributed to heterosis decay, additive fixation, or a genetic trend. Crossbreeding and diallel studies demonstrate that heterosis, general and specific combining ability, reciprocal effects, and maternal influences may contribute differently to growth traits (21, 22). Negash et al. (23) further showed that purebred, general combining-ability, maternal, specific combining-ability, and heterotic effects can contribute simultaneously to crossbred growth performance. Reciprocal combinations involving the genetic backgrounds of CARI Nirbheek and CARI Shyama have also shown differences in production and egg-quality traits (25). The present study, however, did not include parental means, reciprocal crosses, or estimates of additive and dominance effects, and sire–dam family links were unavailable for the analytical dataset. Accordingly, neither heterosis nor individual genetic components could be quantified directly.

Evidence from experimental intercross populations reinforces this cautious interpretation. Adomako et al. (29) reported genotype-dependent differences in body weight, gain, and linear measurements in an F_2_ population produced by inter se mating selected F_1_ crossbreds, while Siegel et al. (30) demonstrated changing phenotypic patterns across recombinant generations extending from F_2_ to F_17_. Animal-model studies in Dahlem Red, native and synthetic chicken populations likewise demonstrate the importance of estimating additive, maternal, permanent environmental, and other variance components before inferring genetic response or population stabilization (31–34). Therefore, the present F_1_-F_2_ comparisons should be regarded as phenotypic contrasts between successive production cohorts rather than estimates of heterosis retention, genetic fixation, or directional selection response.

Egg-weight differences between CARI Nirbheek and CARI Shyama were comparatively modest and age-dependent. In F_1_, CARI Shyama produced slightly heavier eggs at 27 weeks, whereas CARI Nirbheek had slightly heavier eggs at 40 weeks; neither corresponding comparison was significant in F_2_. This change should not be interpreted as biological convergence because egg weight is affected by age, body weight, reproductive maturity, genetic background, and environmental conditions (31, 43, 44). Earlier work involving CARI Nirbheek and CARI Shyama also demonstrated differences in egg weight and other egg-quality characteristics depending on genetic background and cross direction (25). Similar genotype-dependent variation in egg weight has been reported among Nicobari × Vanaraja crosses (16) and among crossbred populations developed for rural poultry production (19). At the parental-breed level, Kumar et al. (45) reported mean egg weights of 41.70 g in Aseel and 40.59 g in Kadaknath under backyard rearing, illustrating that the parental breeds themselves show only modest separation in this trait. More broadly, heterosis, combining ability, reciprocal effects, and maternal effects can all contribute to egg-trait variation in crossbred chickens (24). The present results therefore indicate small, age-dependent line differences in egg weight rather than evidence of loss or stabilization of line-associated variation.

The within-line F_1_-F_2_ comparisons further showed that cohort differences were trait dependent. At 20 weeks, body weight was almost unchanged between cohorts in CARI Shyama, whereas CARI Nirbheek showed a modest 3.8% reduction in F_2_. In contrast, substantially larger F_1_-F_2_ differences occurred at several intermediate growth ages, while morpho-conformation and egg-weight means remained comparatively similar across cohorts. This contrast suggests that intermediate growth measurements were more sensitive to differences between production cycles than the measured conformation and egg-weight traits. Such environmental sensitivity is relevant to rural-poultry evaluation: Rajkumar et al. (46) reported differences in growth and production performance of the same crossbred population when evaluated under farm and backyard conditions, illustrating the influence of production environment on expressed performance. In the present study, F_1_ and F_2_ were produced in different hatching and rearing cycles, so generation was confounded with year, season, batch, and selection history. Consequently, the observed F_1_-F_2_ similarities or differences should be interpreted as cohort-level phenotypic patterns. The comparatively similar conformation and egg-weight means across the two cohorts are encouraging from a phenotypic perspective, but establishing genetic stability would require evaluation over additional inter se generations together with pedigree- or genomic-based estimation of additive and non-additive genetic variance.

### 4.1 Practical implications and limitations

The contrasting phenotypic profiles of CARI Nirbheek and CARI Shyama may have different practical relevance depending on the production objective. The greater later juvenile body weight and larger body-frame profile of CARI Nirbheek may be advantageous where growth and body size are prioritized. For backyard and semi-intensive poultry programmes, however, line choice should consider the intended balance among growth, body conformation, egg performance, adaptability, farmer preference, and local production conditions rather than relying on a single trait (6, 47–49). The present findings may therefore help identify candidate traits for continued evaluation, but extrapolation to farmer-level production systems requires validation across multiple production cycles and under representative low-input and backyard environments. This requirement is supported by Rajkumar et al. (46), who reported differences in laying-period body weight, age at sexual maturity, egg weight, and egg production when the same three-way rural-poultry cross was evaluated under farm and backyard conditions. Such findings emphasize the importance of production environment when extending institutional performance data to field conditions.

Future selection in F_3_ and subsequent generations should be supported by estimation of heritabilities, genetic correlations, genetic (co)variances, and breeding values for the traits of interest. Appropriate economic weights could subsequently be incorporated into a multi-trait selection index to balance later juvenile body weight, body conformation, and relevant egg traits according to the breeding objective. Until such genetic parameters are available, the present phenotypic results should be regarded as baseline information for identifying candidate traits rather than as a basis for a formal selection index.

Breed-level evidence nevertheless indicates that these traits may possess useful genetic variation. Haunshi et al. (41) reported moderate-to-high heritability estimates for juvenile body weight and measurable heritability for shank length in Aseel and Kadaknath, together with strong associations between body weight and shank length. These findings support the potential responsiveness of juvenile growth and conformation traits to selection in the parental breeds, but corresponding genetic parameters should be estimated directly in CARI Nirbheek and CARI Shyama before they are incorporated into a formal breeding strategy.

## 5 Conclusions

This study characterized the phenotypic performance of CARI Nirbheek and CARI Shyama across the foundation cross (F_1_) and first inter se (F_2_) cohorts under institutional management. CARI Nirbheek exhibited greater later juvenile body weight and a larger body-frame profile, with greater shank length, keel length, and breast angle across the measured ages. Egg-weight differences between the lines were modest and age-dependent, with significant differences observed in F_1_ but not in F_2_. Within-line comparisons showed comparatively similar mean values for morpho-conformation and egg-weight traits across the F_1_ and F_2_ cohorts. At 20 weeks, body weight was virtually unchanged between cohorts in CARI Shyama, whereas CARI Nirbheek showed a modest 3.8% reduction in F_2_; larger cohort-level differences were observed at several intermediate growth ages. Because the two cohorts were produced in different hatching and rearing cycles, generation effects could not be separated from year, season, batch, and selection history. Accordingly, these findings represent phenotypic comparisons across cohorts rather than direct evidence of genetic change, heterosis retention, genetic fixation, or true-breeding stability. The observed line-associated phenotypic differences provide baseline information for continued evaluation of growth, body conformation, and egg traits in subsequent generations. Establishing genetic stability and developing reliable selection strategies will require evaluation across successive generations together with pedigree- or genomic-based estimation of additive and non-additive genetic variance and other relevant genetic parameters.

## Data Availability

The phenotypic datasets generated and analyzed during the present study, including body weight, body-weight gain, conformation traits, and egg weight records, are available from the corresponding author upon reasonable request.

## References

[1] Singh M Patton RN Mollier RT Pongener N Yadav R Singh V . Indigenous chicken production system in different agro-ecology of Indian Himalayan region: implication on food and economic security. Front Nutr. (2023) 10:1244413. doi: 10.3389/fnut.2023.1244413 37743914 PMC10512463

[2] Department of Animal Husbandry and Dairying. Basic Animal Husbandry Statistics 2025. New Delhi: Ministry of Fisheries, Animal Husbandry and Dairying, Government of India (2025).

[3] Sharma R Saran S Tyagi JS. Indian poultry sector: strategies for nutritional, socio-economic and environmental security. Indian J Poult Sci. (2023) 58:89–97. doi: 10.5958/0974-8180.2023.00017.X

[4] Department of Animal Husbandry and Dairying. 20th Livestock Census-2019: All India Report. New Delhi: Ministry of Fisheries, Animal Husbandry and Dairying, Government of India (2019).

[5] Desta TT. Sustainable intensification of indigenous village chicken production system: matching the genotype with the environment. Trop Anim Health Prod. (2021) 53:337. doi: 10.1007/s11250-021-02773-5 34021847

[6] Padhi MK. Importance of indigenous breeds of chicken for rural economy and their improvements for higher production performance. Scientifica. (2016) 2016:2604685. doi: 10.1155/2016/2604685 27144053 PMC4838803

[7] Kanakachari M Rahman H Chatterjee RN Bhattacharya TK. Signature of Indian native chicken breeds: a perspective. World's Poult Sci J. (2022) 78:421–45. doi: 10.1080/00439339.2022.2026201

[8] Chatterjee RN Sharma RP Reddy MR Niranjan M Reddy BLN. Growth, body conformation and immunoresponsiveness in two Indian native chicken breeds. Livest Res Rural Dev. (2007) 19:151.

[9] Haunshi S Niranjan M Shanmugam M Padhi MK Reddy MR Sunitha R . Characterization of two Indian native chicken breeds for production, egg and semen quality, and welfare traits. Poult Sci. (2011) 90:314–20. doi: 10.3382/ps.2010-01013 21248327

[10] Rajkumar U Haunshi S Paswan C Raju MVLN Rama Rao SV Chatterjee RN. Characterization of indigenous Aseel chicken breed for morphological, growth, production, and meat composition traits from India. Poult Sci. (2017) 96:2120–6. doi: 10.3382/ps/pew492 28339770

[11] Haunshi S Prince LLL. Kadaknath: a popular native chicken breed of India with unique black colour characteristics. World's Poult Sci J. (2021) 77:427–40. doi: 10.1080/00439339.2021.1897918

[12] Pathak P Dubey PP Dash SK Chaudhary ML. Studies on growth and carcass traits of Aseel and Kadaknath chicken. Indian J Poult Sci. (2015) 50:327–8.

[13] Haunshi S Padhi MK Niranjan M Rajkumar U Shanmugam M Chatterjee RN. Comparative evaluation of native breeds of chicken for persistency of egg production, egg quality and biochemical traits. Indian J Anim Sci. (2013) 83:59–62. doi: 10.56093/ijans.v83i1.26442

[14] Khan AG. Indigenous breeds, crossbreds and synthetic hybrids with modified genetic and economic profiles for rural family and small scale poultry farming in India. World's Poult Sci J. (2008) 64:405–15. doi: 10.1017/S0043933908000135

[15] Niranjan M Sharma RP Rajkumar U Reddy BLN Chatterjee RN Bhattacharya TK. Comparative evaluation of production performance in improved chicken varieties for backyard farming. Int J Poult Sci. (2008) 7:1128–31. doi: 10.3923/ijps.2008.1128.1131

[16] Kundu A De AK Kundu MS Sunder J Jeyakumar S Sujatha T. Production performance of indigenous Nicobari fowls, Vanaraja and their various F_1_ crosses under hot humid climate of Andaman and Nicobar Islands, India. Indian J Anim Sci. (2015) 85:172–7. doi: 10.56093/ijans.v85i2.46611

[17] Padhi MK Chatterjee RN Haunshi S Rajkumar U Niranjan M Rajaravindra KS. Evaluation of four different crossbreds developed for backyard poultry farming under intensive system. Indian J Anim Sci. (2015) 85:985–90. doi: 10.56093/ijans.v85i9.51730

[18] Padhi MK Chatterjee RN Rajkumar U Niranjan M Haunshi S. Evaluation of a three-way cross chicken developed for backyard poultry in respect to growth, production and carcass quality traits under intensive system of rearing. J Appl Anim Res. (2016) 44:390–4. doi: 10.1080/09712119.2015.1091336

[19] Rajaravindra KS Kour A Prince LLL Haunshi S Niranjan M Jayakumar S . Development of novel egg type chicken variety with improved productivity for rural backyards of India. Indian J Anim Sci. (2024) 94:342–9. doi: 10.56093/ijans.v94i4.146229

[20] Yashveer V Nirala R Sinha RRK Bhadauria P Kumar D. Evaluation of growth traits of Aseel, Kadaknath, Dahlem Red and their crosses in the agro climatic conditions of Bihar. Indian J Anim Res. (2025). doi: 10.18805/IJAR.B-5194

[21] Siwendu NA Norris D Ngambi JW Shimelis HA Benyi K. Heterosis and combining ability for body weight in a diallel cross of three chicken genotypes. Trop Anim Health Prod. (2013) 45:965–70. doi: 10.1007/s11250-012-0317-8 23151822

[22] Sungkhapreecha P Chankitisakul V Duangjinda M Boonkum W. Combining abilities, heterosis, growth performance, and carcass characteristics in a diallel cross from black-bone chickens and Thai native chickens. Animals. (2022) 12:1602. doi: 10.3390/ani12131602 35804500 PMC9264945

[23] Negash F Abegaz S Tadesse Y Jembere T Esatu W Dessie T. Estimation of crossbreeding parameters for growth and feed efficiency traits in exotic chicken breed crosses with Fayoumi under reciprocal mating. Sci Afr. (2023) 21:e01851. doi: 10.1016/j.sciaf.2023.e01851

[24] Mokoena K Mbazima V Tyasi TL. A literature review on effect of chicken crossbreeding on egg production traits. World's Poult Sci J. (2025) 81:151–67. doi: 10.1080/00439339.2024.2443184

[25] Prakash JV Kumar K Pandey Y Khare RK. Egg production and egg quality characteristics in direct and reciprocal crosses using CARI Nirbheek and CARI Shyama. J Anim Res. (2020) 10:579–84. doi: 10.30954/2277-940X.04.2020.16

[26] Kour A Haunshi S Rajaravindra KS Prince LLL Rajkumar U. Poultry breeding under public sector in India – achievements and future perspectives: a comprehensive review. Indian J Anim Sci. (2024) 94:191–202. doi: 10.56093/ijans.v94i3.141272

[27] Khadda BS Lata K Kumar R Jadav JK Singh B Palod J. Production performance and economics of CARI Nirbheek chicken for backyard farming under semi-arid ecosystem in Central Gujarat, India. Indian J Anim Res. (2017) 51:382–86. doi: 10.18805/ijar.8421

[28] Pal S Gupta T Wagh S Yadav DK. Comparisons of growth performance of direct and reciprocal crosses of Aseel Peela (AP) and Kadaknath (KN) with CARI-Red. J Entomol Zool Stud. (2019) 7:1356–9.

[29] Adomako K Olympio OS Hagan JK Hamidu JA. Growth performance of crossbred naked neck and normal feathered laying hens kept in tropical villages. Br Poult Sci. (2014) 55:701–8. doi: 10.1080/00071668.2014.960805 25192492

[30] Siegel PB Honaker CF Andersson L. Research note: phenotypic trends for the multigenerational advanced intercross of the Virginia body weight lines of chickens. Poult Sci. (2024) 103:103480. doi: 10.1016/j.psj.2024.103480 38330887 PMC10864792

[31] Tongsiri S Jeyaruban GM Hermesch S van der Werf JHJ Li L Chormai T. Genetic parameters and inbreeding effects for production traits of Thai native chickens. Asian-Australas J Anim Sci. (2019) 32:930–8. doi: 10.5713/ajas.18.0690 30744369 PMC6601067

[32] Prince LLL Rajaravindra KS Rajkumar U Reddy BLN Paswan C Haunshi S . Genetic analysis of growth and egg production traits in synthetic colored broiler female line using animal model. Trop Anim Health Prod. (2020) 52:3153–63. doi: 10.1007/s11250-020-02340-4 32617799

[33] Rajkumar U Prince LLL Rajaravindra KS Haunshi S Niranjan M Chatterjee RN. Analysis of (co)variance components and estimation of breeding value of growth and production traits in Dahlem Red chicken using pedigree relationship in an animal model. PLoS ONE. (2021) 16:e0247779. doi: 10.1371/journal.pone.0247779 33657155 PMC7928508

[34] Chomchuen K Tuntiyasawasdikul V Chankitisakul V Boonkum W. Genetic evaluation of body weights and egg production traits using a multi-trait animal model and selection index in Thai native synthetic chickens (Kaimook e-San2). Animals. (2022) 12:335. doi: 10.3390/ani12030335 35158657 PMC8833322

[35] Das AK Mishra AK Kumar S Rahim A Kokate LS. Characterizing grower performance, body conformation and morphology in crosses of RIR and Indian native chicken genotypes. Indian J Anim Sci. (2017) 87:782–5. doi: 10.56093/ijans.v87i6.71341

[36] Sahoo DP Behura NC Samal L Nandi B Bagh J Nayak GD . Growth performance, feed efficiency and linear body measurements of triple cross progenies of Hansli, CSML and CSFL chickens. e-planet. (2017) 15:127–32.

[37] Wickham H François R Henry L Müller K Vaughan D. dplyr: A Grammar of Data Manipulation. R package (2026).

[38] Schauberger P Walker A. openxlsx: Read, Write and Edit xlsx Files. R package (2025).

[39] Moharrery A Mirzaei M. Growth characteristics of commercial broiler and native chickens as predicted by different growth functions. J Anim Feed Sci. (2014) 23:82–9. doi: 10.22358/jafs/65720/2014

[40] González Ariza A Arando Arbulu A Navas González FJ Nogales Baena S Delgado Bermejo JV Camacho Vallejo ME. The study of growth and performance in local chicken breeds and varieties: a review of methods and scientific transference. Animals. (2021) 11:2492. doi: 10.3390/ani11092492 34573456 PMC8466095

[41] Haunshi S Shanmugam M Padhi MK Niranjan M Rajkumar U Reddy MR . Evaluation of two Indian native chicken breeds for reproduction traits and heritability of juvenile growth traits. Trop Anim Health Prod. (2012) 44:969–73. doi: 10.1007/s11250-011-9994-y 22068634

[42] Kumar M Dahiya SP Ratwan P Sheoran N. Evaluation of morphological traits in Aseel and Kadaknath breeds under backyard poultry farming using principal component analysis. Turk J Vet Anim Sci. (2022) 46:356–65. doi: 10.55730/1300-0128.4183

[43] Mookprom S Boonkum W Kunhareang S Siripanya S Duangjinda M. Genetic evaluation of egg production curve in Thai native chickens by random regression and spline models. Poult Sci. (2017) 96:274–81. doi: 10.3382/ps/pew326 27665013

[44] Chandan P Bhattacharya TK Rajkumar U Prince LLL Chatterjee RN. Estimation of genetic parameters of growth and egg production traits by animal model in IWK layer strain. Indian J Anim Res. (2019) 53:1252–7. doi: 10.18805/ijar.B-3638

[45] Kumar M Dahiya SP Ratwan P Sheoran N Kumar S Kumar N. Assessment of egg quality and biochemical parameters of Aseel and Kadaknath indigenous chicken breeds of India under backyard poultry farming. Poult Sci. (2022) 101:101589. doi: 10.1016/j.psj.2021.101589 34933223 PMC8693008

[46] Rajkumar U Haunshi S Paswan C Reddy BLN Yadav SP. Evaluation of a three-way crossbred chicken developed for rural poultry under farm and backyard conditions for growth and production traits. Indian J Anim Sci. (2018) 88:229–32. doi: 10.56093/ijans.v88i2.79348

[47] Pica-Ciamarra U Otte J. Poultry, food security and poverty in India: looking beyond the farm-gate. World's Poult Sci J. (2010) 66:309–20. doi: 10.1017/S0043933910000358

[48] Kumar M Dahiya SP Ratwan P. Backyard poultry farming in India: a tool for nutritional security and women empowerment. Biol Rhythm Res. (2021) 52:1476–91. doi: 10.1080/09291016.2019.1628396

[49] Rajkumar U Rama Rao SV Raju MVLN Chatterjee RN. Backyard poultry farming for sustained production and enhanced nutritional and livelihood security with special reference to India: a review. Trop Anim Health Prod. (2021) 53:176. doi: 10.1007/s11250-021-02621-6 33611639

