# Supplementary material for: Growth, conformation, and egg-weight traits of CARI Nirbheek and CARI Shyama chickens across the foundation cross and first inter se generation
Source: Front Vet Sci. 2026 Oct 8;13:1945142. doi: 10.3389/fvets.2026.1945142 (PMC13686922; doi:10.3389/fvets.2026.1945142)
Supplement: Supplementary file 1 [file Data_Sheet_1.pdf]

Within-line phenotypic comparison of F_1 and F_2 cohorts in CARI Nirbheek and CARI Shyama

Within-line comparison of the foundation cross (F_1) and first inter se cohort (F_2) for body weight, morpho-conformation traits, and egg weight. Differences were evaluated using Welch's two-sample t-test. For these supplementary within-line cohort comparisons, the 46 F_1–F_2 contrasts were adjusted jointly using the Benjamini–Hochberg false-discovery-rate procedure. Standard deviations were reconstructed as SE × √n (body weight: n = 720 [F_1] and 600 [F_2] per line; morpho-conformation: n = 400 per line per cohort; egg weight: n = 100 per line per cohort). Percentage change was calculated as $100 \times (F_2 - F_1)/F_1$. Cohen's d was calculated as $(F_1 - F_2)$/pooled SD. Adjusted $P < 0.05$ was considered significant.

Table S1. Body weight (g) - F_1 vs F_2 within line

Age	Line	F1 (mean ± SE)	F2 (mean ± SE)	% change	d	P (BH-FDR)
Day-old	CARI Nirbheek	32.61 ± 0.143	32.57 ± 0.155	-0.1	0.01	1.0000 ns
Day-old	CARI Shyama	30.76 ± 0.118	32.62 ± 0.447	+6.0	-0.24	<0.0001 ***
2 wk	CARI Nirbheek	70.09 ± 0.449	77.83 ± 0.351	+11.0	-0.73	<0.0001 ***
2 wk	CARI Shyama	70.12 ± 0.337	71.03 ± 0.328	+1.3	-0.11	0.0874 ns
4 wk	CARI Nirbheek	144.35 ± 1.493	151.05 ± 0.423	+4.6	-0.22	<0.0001 ***
4 wk	CARI Shyama	149.78 ± 1.845	157.29 ± 0.311	+5.0	-0.20	<0.0001 ***
6 wk	CARI Nirbheek	219.79 ± 1.838	264.56 ± 0.995	+20.4	-1.12	<0.0001 ***
6 wk	CARI Shyama	226.73 ± 1.861	264.52 ± 0.468	+16.7	-1.00	<0.0001 ***
8 wk	CARI Nirbheek	403.37 ± 3.048	435.28 ± 1.820	+7.9	-0.47	<0.0001 ***
8 wk	CARI Shyama	350.26 ± 2.421	357.31 ± 0.958	+2.0	-0.14	0.0099 **
10 wk	CARI Nirbheek	446.60 ± 3.323	616.37 ± 1.556	+38.0	-2.40	<0.0001 ***
10 wk	CARI Shyama	402.18 ± 3.069	467.44 ± 0.547	+16.2	-1.06	<0.0001 ***
12 wk	CARI Nirbheek	631.52 ± 4.226	940.49 ± 1.325	+48.9	-3.57	<0.0001 ***

Age	Line	F1 (mean ± SE)	F2 (mean ± SE)	% change	d	P (BH-FDR)
12 wk	CARI Shyama	598.66 ± 8.402	899.50 ± 2.669	+50.3	-1.75	<0.0001 ***
16 wk	CARI Nirbheek	921.73 ± 2.986	1085.79 ± 8.537	+17.8	-1.07	<0.0001 ***
16 wk	CARI Shyama	899.20 ± 11.198	904.30 ± 6.196	+0.6	-0.02	1.0000 ns
20 wk	CARI Nirbheek	1285.19 ± 6.383	1236.66 ± 8.367	-3.8	0.26	<0.0001 ***
20 wk	CARI Shyama	1178.94 ± 9.079	1187.57 ± 10.368	+0.7	-0.03	1.0000 ns

Table S2. Shank length (mm) - F_1 vs F_2 within line

Age	Line	F1 (mean ± SE)	F2 (mean ± SE)	% change	d	P (BH-FDR)
6 wk	CARI Nirbheek	39.07 ± 0.236	39.74 ± 0.226	+1.7	-0.14	0.0631 ns
6 wk	CARI Shyama	34.30 ± 0.140	34.39 ± 0.142	+0.3	-0.03	1.0000 ns
10 wk	CARI Nirbheek	58.17 ± 0.298	57.96 ± 0.285	-0.4	0.04	1.0000 ns
10 wk	CARI Shyama	41.96 ± 0.112	41.95 ± 0.112	0.0	0.00	1.0000 ns
14 wk	CARI Nirbheek	84.34 ± 0.135	84.28 ± 0.134	-0.1	0.03	1.0000 ns
14 wk	CARI Shyama	74.24 ± 0.164	74.30 ± 0.161	+0.1	-0.02	1.0000 ns
20 wk	CARI Nirbheek	91.61 ± 0.133	91.56 ± 0.132	-0.1	0.02	1.0000 ns
20 wk	CARI Shyama	83.00 ± 0.184	83.01 ± 0.171	+0.0	-0.00	1.0000 ns

Table S3. Keel length (mm) - F_1 vs F_2 within line

Age	Line	F1 (mean ± SE)	F2 (mean ± SE)	% change	d	P (BH-FDR)
6 wk	CARI Nirbheek	44.53 ± 0.155	44.49 ± 0.160	-0.1	0.01	1.0000 ns
6 wk	CARI Shyama	43.10 ± 0.130	43.01 ± 0.131	-0.2	0.04	1.0000 ns
10 wk	CARI Nirbheek	66.84 ± 0.293	66.15 ± 0.289	-1.0	0.12	0.1517 ns
10 wk	CARI Shyama	64.25 ± 0.207	64.24 ± 0.212	-0.0	0.00	1.0000 ns
14 wk	CARI Nirbheek	80.18 ± 0.168	80.13 ± 0.170	-0.1	0.02	1.0000 ns
14 wk	CARI Shyama	78.08 ± 0.124	77.97 ± 0.122	-0.1	0.05	1.0000 ns
20 wk	CARI Nirbheek	99.48 ± 0.168	99.43 ± 0.170	-0.1	0.02	1.0000 ns
20 wk	CARI Shyama	93.91 ± 0.141	93.85 ± 0.135	-0.1	0.02	1.0000 ns

Table S4. Breast angle (°) - F_1 vs F_2 within line

Age	Line	F1 (mean ± SE)	F2 (mean ± SE)	% change	d	P (BH-FDR)
6 wk	CARI Nirbheek	25.09 ± 0.163	24.64 ± 0.152	-1.8	0.15	0.0566 ns
6 wk	CARI Shyama	23.73 ± 0.187	23.42 ± 0.185	-1.3	0.09	0.4011 ns
10 wk	CARI Nirbheek	39.01 ± 0.129	38.76 ± 0.138	-0.7	0.10	0.2762 ns
10 wk	CARI Shyama	37.17 ± 0.125	37.20 ± 0.120	+0.1	-0.01	1.0000 ns
14 wk	CARI Nirbheek	47.37 ± 0.065	47.40 ± 0.066	+0.0	-0.02	1.0000 ns
14 wk	CARI Shyama	45.39 ± 0.097	45.64 ± 0.089	+0.6	-0.14	0.0748 ns
20 wk	CARI Nirbheek	60.09 ± 0.122	59.95 ± 0.129	-0.2	0.06	0.7926 ns

Age	Line	F1 (mean ± SE)	F2 (mean ± SE)	% change	d	P (BH-FDR)
20 wk	CARI Shyama	57.81 ± 0.058	57.86 ± 0.057	+0.1	-0.05	1.0000 ns

Table S5. Egg weight (g) - F_1 vs F_2 within line

Age	Line	F1 (mean ± SE)	F2 (mean ± SE)	% change	d	P (BH-FDR)
27 wk (recording age)	CARI Nirbheek	39.89 ± 0.318	39.91 ± 0.322	+0.1	-0.01	1.0000 ns
27 wk (recording age)	CARI Shyama	41.03 ± 0.316	40.69 ± 0.314	-0.8	0.11	0.9108 ns
40 wk (recording age)	CARI Nirbheek	51.03 ± 0.150	50.93 ± 0.190	-0.2	0.06	1.0000 ns
40 wk (recording age)	CARI Shyama	50.54 ± 0.181	50.50 ± 0.219	-0.1	0.02	1.0000 ns

Significance: *** P < 0.001, ** P < 0.01, * P < 0.05, ns = not significant (all adjusted).
